# The effects of blood flow restriction training on post activation potentiation and upper limb muscle activation: a meta-analysis

**DOI:** 10.3389/fphys.2024.1395283

**Published:** 2024-07-11

**Authors:** Haiyang Liu, Lizhu Jiang, Jian Wang

**Affiliations:** ^1^ Department of Physical Education, Ningbo University of Technology, Ningbo, China; ^2^ Ningde Vocational and Technical College, Ningde, China

**Keywords:** blood flow restriction training, muscle activation, post-activation potentiation, meta-analysis, lower limb muscle

## Abstract

**Objective:**

This meta-analysis aims to systematically evaluate the impact of blood flow restriction training (BFRT) on muscle activation and post-activation potentiation (PAP) in the upper limbs, to provide guidance for upper limb protocols aiming to enhance explosive strength and activation.

**Methods:**

PubMed, CNKI, Web of Science, and EBSCO databases were queried to identify randomized controlled trials (RCTs) investigating the effects of upper limb BFRT on muscle activation and PAP. Inclusion and exclusion criteria were applied using the Cochrane bias risk tool. Literature quality assessment and statistical analysis were conducted using Revman 5.4 and Stata 17.0 software. Sensitivity analysis and funnel plots were utilized to assess result stability and publication bias.

**Results:**

A total of 31 articles involving 484 participants were included in the analysis. Meta-analysis results showed that upper limb BFRT significantly increased muscle iEMG values [*SMD* = 0.89, 95%*CI* (0.21, 1.58), *p* = 0.01]. BFRT had a significant effect on upper limb explosive force [*SMD* = 0.73, 95%*CI* (0.41, 1.04), *p* < 0.00001]. Subgroup analysis based on literature heterogeneity (*I* ^2^ = 92%, 80%) showed that exhaustive BFRT significantly decreased upper limb iEMG [*SMD* = −0.67, 95%*CI* (−1.25, −0.09), *p* = 0.01], with exercise modes including maximum output power of bench press [*SMD* = 1.87, 95%*CI* (0.22, 3.53), *p* < 0.0001], exercise intensity of 40%–70% 1RM [*SMD* = 1.31, 95%*CI* (0.61, 2.01), *p* < 0.0001], and pressure intensity of ≥60% AOP [*SMD* = 0.83, 95%*CI* (0.43, 1.23), *p* < 0.0001] reaching maximum effects and statistical significance.

**Conclusion:**

Upper limb BFRT can induce muscle activation and PAP. BFRT with 40%–70% 1RM and ≥60% AOP in the upper limbs is more likely to promote PAP.

**Systematic Review Registration:**

http://inplasy.com, identifier INPLASY202430008.

## 1 Introduction

With increasing competitiveness in sports, conventional training methodologies frequently lag in fulfilling athletes’ requisites for augmenting their competitive prowess (McGuigan et al., 2012).Improving muscle explosiveness represents a prevalent objective among athletes engaged in disciplines such as throwing, jumping, and various other sports.

Muscle activation and post-activation potentiation (PAP) are considered important mechanisms for improving strength and explosiveness (Sevilmi and Atala, 2019; Monteiro-Oliveira et al., 2022). Muscle activation represents the responsiveness of the muscle nervous system to movement tasks (Pourmoghaddam et al., 2016), with higher muscle activation implying more muscle fibers involved in the movement, thereby enhancing strength and explosiveness (Wang, 2021). As a means to rapidly enhance strength, post-activation potentiation (PAP) is attained through controlled training exercises like squats and deadlifts. These activities trigger intense neuromuscular excitement, leading to a swift improvement in muscle explosiveness within a concise timeframe (Batista Mauro et al., 2011). This effect is primarily due to increased excitability of the neuromuscular system, leading to enhanced muscle fiber contraction capacity (Wilson et al., 2013). Research shows that post-activation potentiation can optimize athletes’ warm-up routines and enhance athletic performance (Kellis et al., 2015). Blood flow restriction training (BFR) is a method of strength training that involves applying pressure to the human limbs using pressure cuffs, which can block or limit blood flow in the veins or arteries of the limbs. Past studies have shown that BFR training combined with 30% 1RM exercise loads can achieve similar muscle improvement effects to traditional high-load training, providing a safer and more effective option for athlete training (Grnfeldt et al., 2020).

As an emerging training method, blood flow restriction training (BFRT) has attracted increasing attention. A recent meta-analysis found that BFR training can induce lower extremity muscle activation and PAP (Wang J. et al., 2023). Although research on blood flow restriction training in the lower limbs has made some progress, studies on its application in the upper limbs are relatively scarce (Wortman et al., 2021; Wang et al., 2022). Currently, there is a lack of published research and review literature on the application of BFRT in the upper limbs. Therefore, specific protocols and effects of upper limb BFRT remain to be explored. In light of this, this paper aims to systematically analyze the effects of BFR training on upper limb muscle activation and PAP through meta-analysis, further expanding the application scope of BFRT in sports training, and providing more reliable theoretical and practical guidance for improving athletes’ competitive performance and preventing sports injuries.

## 2 Materials and methods

### 2.1 Search strategy

On 29 January 2024, a total of 2025 articles were retrieved from PubMed, CNKI, Web of Science, and EBSCO databases. The English search terms used were: (“blood flow restriction training” or “BFR” or “KAATSU training” or “pressure training”) and (“Potentiation after activation” or “PAP” or “muscle activation” or “upper limbs” “upper extremities”) and (“RCT”).

### 2.2 Inclusion and exclusion criteria

#### 2.2.1 Inclusion criteria

Research Type: This study focuses on randomized controlled trials (RCTs) that investigate the effects of blood flow restriction (BFR) training on muscle activation and fatigue levels. All articles must be publicly published.

Study Participants: The study includes healthy adult participants, regardless of their prior training experience.

Intervention Measures: The experimental group undergoes blood flow restriction training, while the control group participates in alternative training methods.

Outcome Measures: The study assesses quantitative indicators such as maximum strength, electromyography (EMG) values, 1 repetition maximum (1RM).

Additional Criteria: Each study must provide comprehensive details about the experimental design, including the intensity of the blood flow restriction training and other relevant methodological information.

#### 2.2.2 Exclusion criteria

Unclear research type: Studies that do not clearly document their research type will be excluded.

Non-BFR training: Studies that involve interventions other than blood flow restriction training will be excluded.

Duplicate publications: Articles that are repeatedly published, those for which the full text cannot be obtained, and review articles will be excluded.

Lack of quantitative outcome data: Studies without quantitative outcome indicators or valid data will be excluded.

Animal Experiments: Research involving animal experiments will be excluded.

### 2.3 Data extraction

**FIGURE 1:**
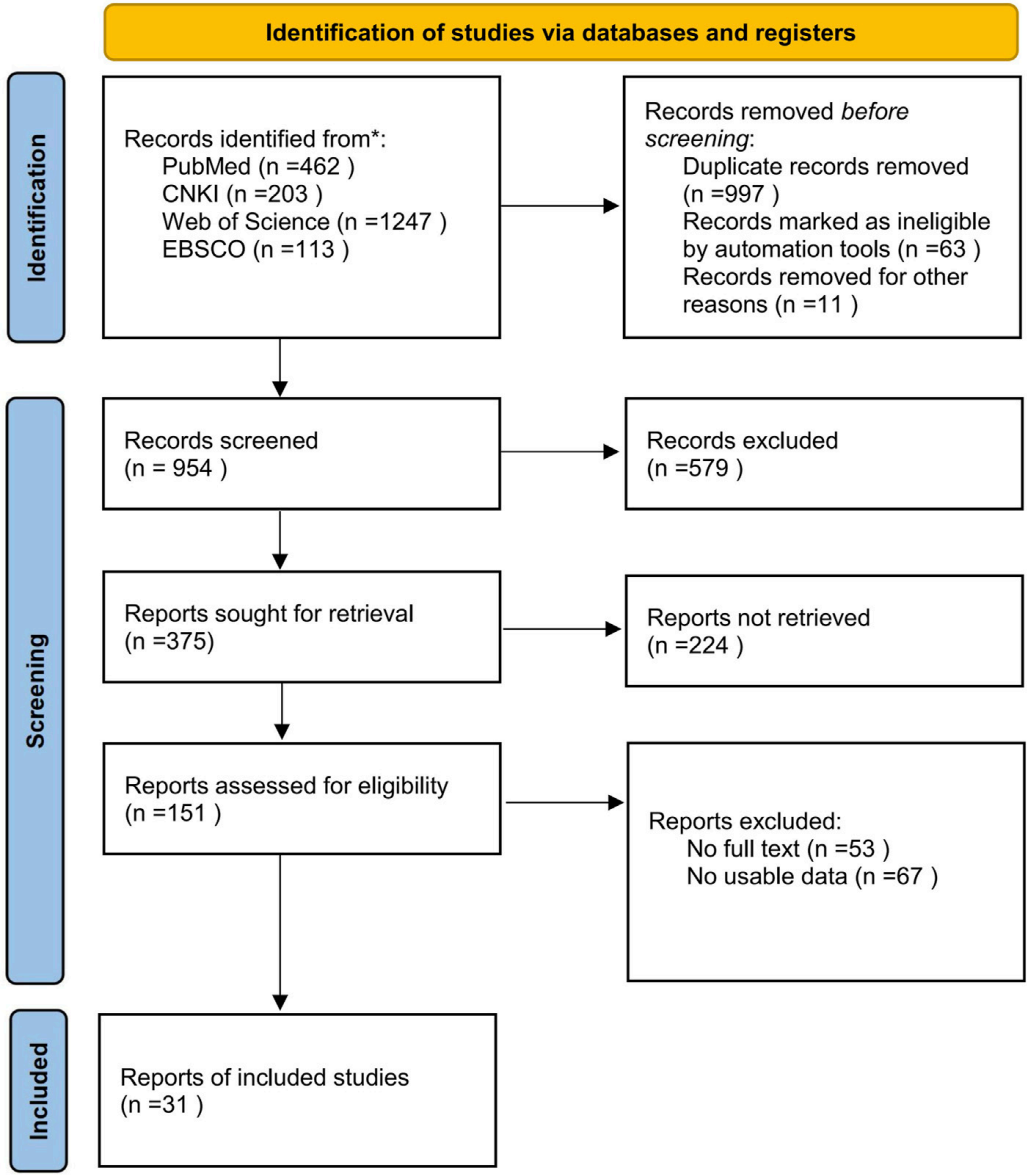
Flow diagram of literature selection.

Literature screening and inclusion steps were conducted using EndNote software, with independent screening by JW and HL. The process is outlined in Figure 1, resulting in the inclusion of 31 papers in the review.

Data Extraction: Information extraction was independently performed by two researchers using a custom-made form, primarily covering the following categories:

1. General Information: First author and publication year.
2. Sample Information: Details about the research subjects, including age, sample sizes for both experimental and control groups.
3. Characteristics of Exercise Intervention: Information on intervention measures for both groups, as well as specifics of the intervention programs for the experimental group (including training methods, volume, and intensity, cuff intensity).
4. Outcome Indicators: Relevant test indicators for upper limb muscle activation and post-activation potentiation (PAP).

### 2.4 Statistical analysis

Statistical analysis was conducted using Review Manager 5.4 software. Continuous variables were the outcome indicators in the literature, and standardized mean differences (*SMD*) with 95% confidence intervals were chosen as effect sizes due to the different testing methods for each indicator. The Cochrane Risk Bias Assessment Tool was utilized for assessing the quality of the literature. A homogeneity test (*Q* test) was performed to assess heterogeneity, with a significance level (α) set at 0.1. The *I* ^*2*^ values, ranging from 0% to 100%, were considered, where an *I* ^2^ value greater than 50% and a *p*-value less than α indicated the presence of heterogeneity, leading to the selection of the random-effects model. Conversely, the fixed-effect model was chosen when heterogeneity was absent. Subgroup analysis was employed to address heterogeneity, and sensitivity analysis using Stata 17.0 was conducted to test result stability. Egger’s test and funnel plot were utilized to assess the presence of publication bias.

## 3 Results

### 3.1 Study characteristics

A total of 31 publications were included in this study, all of which were RCT, including 484 subjects with mixed gender and age range of 16–74 years, with the basic characteristics shown in Table 1.

**TABLE 1:** Characteristic of studies included in systematic review and meta-analysis.

Study	Country	Age (years)	N (EG/CG)	Intervention (EG/CG)	Plan (BFR intensity)	Outcome extracted
Zhang (2023)	China	20.20 ± 0.92	10/10	BFR/No BFR	4 sets of 8 repetitions of bench press at 30% 1RM (140 mmHg)	BP↑ P (W)↑ PV↑
Serrano-Ramon et al. (2023)	Spain	23.6 ± 4.1	14/14	BFR/No BFR	3 repetitions of bench press at 60% 1RM (80% AOP)	BP↑ PV↑
Ahmadi et al. (2015)	Canada	24.7 ± 4.9	13/13	BFR/No BFR	30-s maximal voluntary contraction of elbow flexion (100% AOP)	MVC ↓ EMG BB↓
Zhao (2023)	China	19 ± 1.23	20/20	BFR/No BFR	15–20 repetitions of pull-ups (150 mmHg)	MVC BB↑ RMS BB↑
Jessee et al. (2017)	United States of America	18–35	29/29	BFR/No BFR	4 groups of 30–15–15–15 times 30% 1RM elbow flexors (30% AOP)	MVC↓ EMG↑
Che et al. (2022)	China	23.6 ± 3.1	10/10	BFR/No BFR	4 groups of 30–15–15–15 times 30% 1RM bench press (160 mmHg)	RMS BB↑
Dankel et al. (2017a)	United States of America	18–35	15/15	BFR/No BFR	4 groups of 30–15–15–15 times 30% 1RM elbow flexors (40% AOP, 160 mmHg)	EMG↑ MVC↓
Roehl et al. (2023)	United States of America	29.4 ± 4.3	15/15	BFR/No BFR	3 repetition of common rotator cuff exercises at 1RM (170 mmHg)	EMG ↑
Henrique et al. (2019)	Brazil	23.0 ± 2.67	13/13	BFR/LL	4 sets of 8 repetitions of elbow flexors at 30% 1RM (20 mmHg)	EF↑
Wilk et al. (2020a)	Poland	29.8 ± 4.6	10/10	BFR/No BFR	3 sets of 3 repetitions of bench press at 70% 1RM (60% AOP, 152 ± 11.4 mmHg)	BP↑ P(W)↑ PV↑
Buckner et al. (2018)	United States of America	22 ± 2	22/22	BFR/No BFR	4 sets of elbow flexors to failure at 15% 1RM (40% AOP)	MVC BB↓ EMG↓
Lambert et al. (2014)	United States of America	18–45	16/16	BFR/No BFR	4 groups of 30–15–15–15 times 20% 1RM dumbbell scaption (50% AOP)	EF↑ EMG↑
Lei (2023)	China	23.67 ± 1.73	10/10	BFR/No BFR	3 sets of 8 repetitions of bench press at 70% 1RM (180 mmHg)	RMS BB↑
Dankel et al. (2017b)	United States of America	26 ± 3	10/10	BFR/No BFR	2 sets of elbow flexors to failure at 70% 1RM (70% AOP)	EMG NS
Yasuda et al. (2015a)	Japan	27 ± 5	10/10	BFR/No BFR	4 sets of elbow flexors to failure at 20% 1RM (160 mmHg)	EMG↓
Carla Florianovicz et al. (2020)	Brazil	21 ± 1.67	58/58	BFR/No BFR	10 sets of 6 repetitions of wrist curl at 40% 1RM (140 ± 12.79 mmHg)	GS↑
Wilk et al. (2020b)	Poland	23.2 ± 2.66	12/12	BFR/No BFR	1 repetition of bench press at 1RM (100% AOP, 135 ± 16 mmHg)	BP↑ P(W) NS PV NS
Wilk et al. (2021)	Poland	25 ± 2	10/10	BFR/No BFR	5 sets of 3 repetitions of bench press at 60% 1RM (80% AOP)	P (W)↑ PV↑
Salagas et al. (2022)	Greece	25.8 ± 6	12/12	BFR/No BFR	4 sets of 12-s rapid bench press at 60% 1RM (100% AOP, 146 ± 15 mmHg)	PV↑
Wang et al. (2023b)	China	23.4 ± 3.1	10/10	BFR/No BFR	4 groups of 30–15–15–15 times 25% 1RM elbow flexors (50% AOP)	RMS↑
Rodrigues et al. (2023)	Brazil	29.9 ± 5.9	15/15	BFR/No BFR	1 repetition of bench press at 1RM (170 mmHg)	BP↑
Sun et al. (2020)	China	25.2 ± 4.0	8/8	BFR/No BFR	6 sets of dumbbell curls to failure at 50% 1RM (200 mmHg)	RMS BB↓ EF↑
Lin et al. (2018)	China	21.75 ± 1.75	8/8	BFR/No BFR	1 min local vibration (200 mmHg)	EMG↑
Thiebaud et al. (2014)	United States of America	22.4 ± 3.2	9/9	BFR/No BFR	4 groups of 30–15–15–15 times 30% 1RM elbow flexors (120 mmHg)	MVC BB↑ EMG↑
Mendonca et al. (2018)	Portugal	22.0 ± 2.0	62/62	BFR/No BFR	4 groups of 30–15–15–15 times 20% 1RM elbow flexors (60% AOP, 139 ± 11 mmHg)	MVC BB↑
Wilk et al. (2022)	Poland	27.6 ± 3.5	14/14	BFR/No BFR	4 sets of 3 repetitions of bench press at 70% 1RM (90% AOP, 323 ± 22 mmHg)	P (W)↑ PV↑
Zhang (2021)	China	22.5 ± 2.7	20/20	BFR/No BFR	6 sets of 8 repetitions of elbow flexors at 30% 1RM (110 mmHg)	RMS BB↑ EF↑
Yasuda et al. (2009)	Japan	24.1 ± 3.2	10/10	BFR/No BFR	4 groups of 30–15–15–15 times 20% 1RM elbow flexors (160 mmHg)	EMG↑
Yasuda et al. (2014)	Japan	23–41	9/9	BFR/No BFR	4 groups of 30–15–15–15 times 20% 1RM elbow flexors (170–260 mmHg)	EMG↑
Li et al. (2022)	China	19.7 ± 3.2	10/10	BFR/No BFR	4 groups of 30–15–15–15 times 30% 1RM bench press (160 mmHg)	BP↑
Linero and Choi (2021)	South Korea	56 ± 18	25/25	BFR/No BFR	3 sets of 20 repetitions of bench press at 30% 1RM (152 ± 6 mmHg)	EF↑

NS, no statistical significance; RMS, electromyographic standard value; MVC, maximum voluntary contraction; ↑ represents a significant increase; ↓ represents a significant decrease; BP, maximum strength of bench press; P(W), maximum output power; PV, velocity of bench press; EF, maximum strength of elbow flexors; EMG, integrated electromyography; LL, low load exercise; BB, biceps brachii; GS, grip strength.

### 3.2 Study quality assessment

**FIGURE 2:**
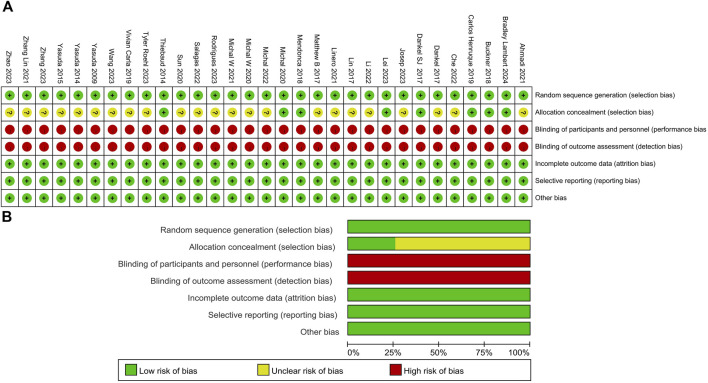
Methodological quality graph and summary of the included studies: **(A)** Risk of bias summary; **(B)** Risk of bias graph.

The quality of the literature was evaluated with reference to the Cochrane Risk of Bias Assessment Tool (Higgins and Altman, 2007). Review Manager 5.4 software assessed seven aspects, including random sequence generation, allocation concealment, participant blinding, outcome blinding, incomplete outcome data, selective reporting, and other bias (Figures 2A,B). Twenty-three articles did not clearly describe whether allocation personnel strictly adhered to random allocation, while 31 articles were at high risk of bias in blinding due to the signing of informed consent forms before the experiment.

### 3.3 Upper limb muscle activation

**FIGURE 3:**
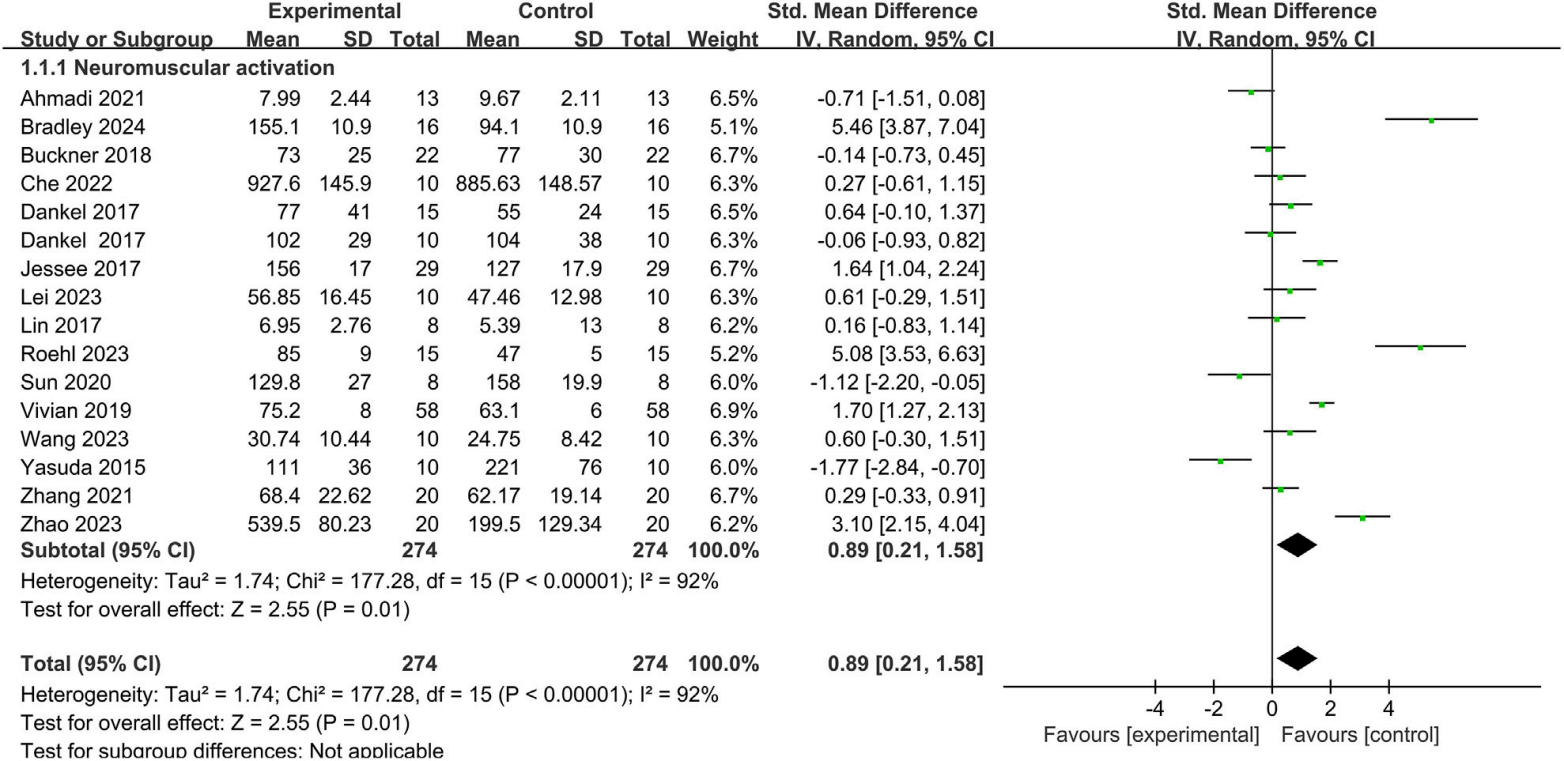
Effect of BFR training on neuromuscular activation.

Among the 31 articles, 16 compared the iEMG before and after BFR training for a total of 274 participants (Figure 3). Upon heterogeneity testing, *I* ^*2*^ was found to be 92% (>50%), and the *Q* test yielded a *p*-value of <0.01, indicating significant heterogeneity among the included studies. Therefore, a random-effects model was chosen for meta-analysis. The results showed a combined effect size of *SMD* = 0.89, which was statistically significant (Z = 2.55, *p* = 0.01 < 0.05). This suggests that compared to the control group, BFR training significantly increased upper limb muscle iEMG values.

### 3.4 PAP

**FIGURE 4:**
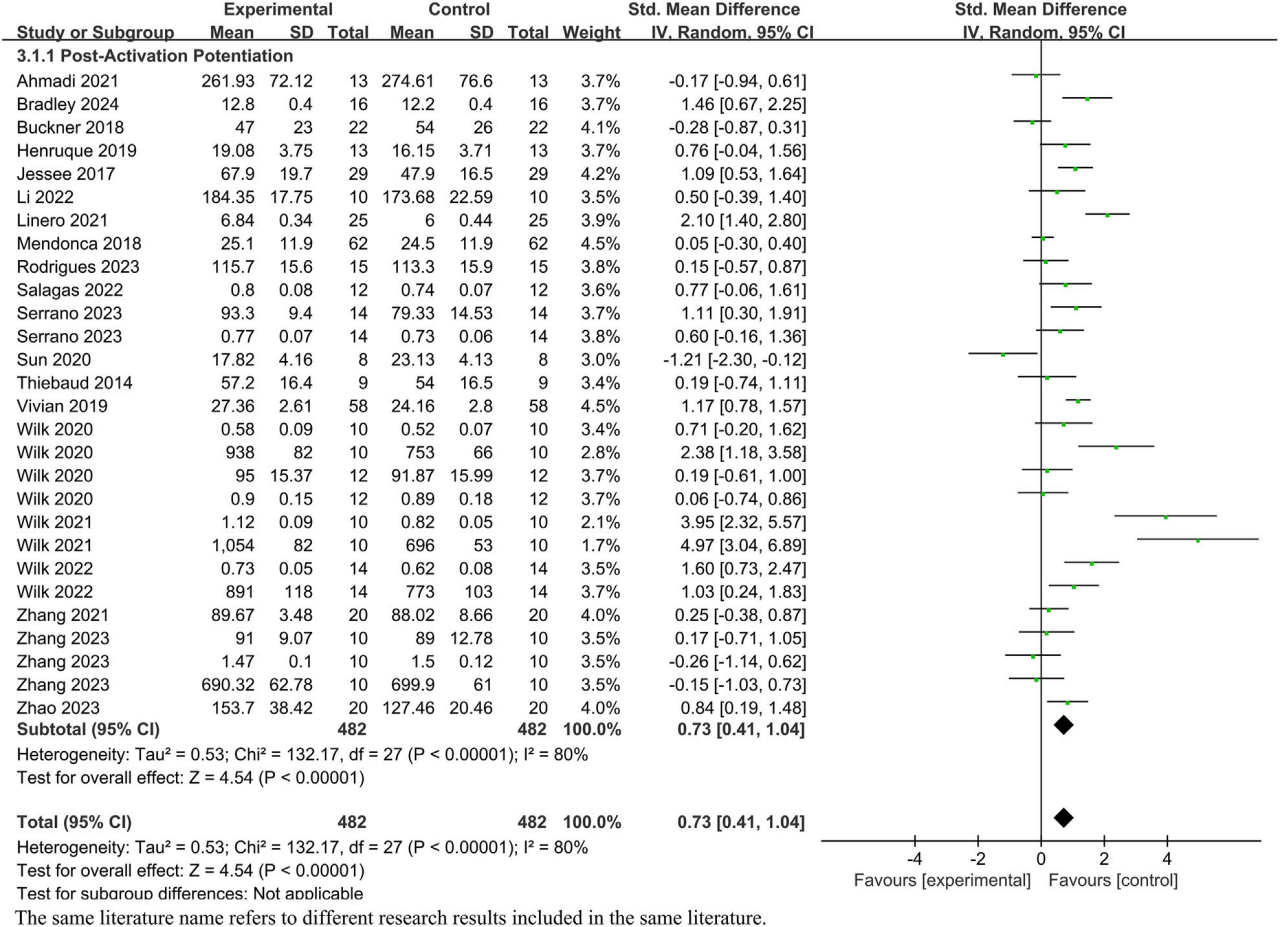
Forest plot of the impact of BFR training on PAP.

Among the 31 articles (Figure 4), a total of 21 were included (comprising 28 studies with 482 participants). The heterogeneity testing showed an *I* ^*2*^ value of 80% (>50%) and a *p*-value of <0.01, indicating significant heterogeneity. Therefore, a random-effects model was employed for meta-analysis. The combined effect size from the 28 studies was *SMD* = 0.73, with a 95% confidence interval of 0.41–1.04, which was statistically significant (*Z* = 4.54, *p* < 0.01). This suggests that upper limb BFR training can induce the occurrence of post-activation potentiation (PAP).

### 3.5 Subgroup analysis

Based on the data from this study, the authors suspect that the source of heterogeneity may be related to exercise mode, exercise intensity, and pressure intensity. Therefore, subgroup analysis was conducted based on the characteristics of the studies mentioned above. The results (Table 2) showed that within different exercise modes, bench press and dumbbell scaption exhibited the highest homogeneity (*I* ^2^ = 0%), significantly reducing heterogeneity compared to the overall combined effect (*I* ^2^ = 92%).

**TABLE 2:** Subgroup analysis of the effects of upper limb BFRT on muscle activation.

Research features	Subgroup standard	Study (sample)	*SMD*	95%*CI*	*P*	I2 (%)	*P* (Heterogeneity)
Exercise mode	Bench press	2 (20)	0.44	−0.19, 1.07	0.17	0	0.60
	Elbow flexors	9 (137)	−0.34	−0.87, 0.18	0.20	76	<0.0001
	Dumbbell scaption	2 (31)	5.26	4.15, 6.37	<0.0001	0	0.74
	Pull-up	1 (20)	3.10	2.15, 4.04	<0.0001	N	N
	Local vibration	1 (8)	0.61	−0.40, 1.62	0.24	N	N
	Wrist curl	1 (58)	1.70	1.27, 2.13	<0.0001	N	N
Exercise intensity	≤30% 1RM	6 (100)	2.06	0.73, 3.39	0.002	93	<0.0001
	40%–70% 1RM	3 (83)	1.04	0.23, 1.86	0.01	77	0.01
	Maximal effort	5 (63)	−0.67	−1.25, −0.09	0.02	57	0.05
	Self weight	2 (28)	1.63	−1.25, 4.51	0.27	94	<0.0001
Compressive strength	≤40% AOP	3 (61)	0.50	−0.73, 1.72	0.43	90	<0.0001
	40%–60% AOP	3 (46)	1.99	−0.34, 4.32	0.09	94	<0.0001
	≥60 AOP	10 (167)	0.75	−0.23, 1.72	0.13	93	<0.0001

Specifically, within exercise intensities of ≤30% 1RM (*I* ^2^ = 93%) and bodyweight resistance (*I* ^2^ = 94%), intragroup heterogeneity increased. Additionally, within pressure intensities of 40%–60% AOP (*I* ^2^ = 94%) and ≥60% AOP (*I* ^2^ = 93%), intragroup heterogeneity also increased. This indicates a strong heterogeneity among studies with exercise intensities of 30% or lower and pressure intensities greater than 40% AOP.

Moreover, studies on pull-ups, local vibration, and wrist curl exercises were limited and lacked representativeness. Subgroup analysis also found that BFR exercises with dumbbell scaption (*SMD* = 5.26) and exercise intensity ≤30% 1RM (*SMD* = 2.06) had better effects on enhancing upper limb muscle electromyography (*p* < 0.05).

Subgroup analysis of the effects of upper limb BFR, training on PAP was conducted based on characteristics that could potentially cause heterogeneity, including testing methods, exercise intensity, and pressure intensity.

The subgroup analysis based on testing methods revealed (Table 3) that bench press exhibited the highest homogeneity (*I*2 = 0%). In comparison to the overall combined effect (*I* ^*2*^ = 80%), higher intragroup heterogeneity was observed for Elbow flexors (*I* ^*2*^ = 88%) and maximum output power (*I* ^*2*^ = 89%). Significance was found in the bench press, velocity of bench press, and maximum output power groups (*p* < 0.05), indicating significant improvements in these indicators due to upper limb BFR training.

**TABLE 3:** Subgroup analysis of upper limb PAP induced by BFR training.

Research features	Subgroup standard	Study (sample)	SMD	95%CI	*P*	I2 (%)	*P* (Heterogeneity)
Testing methods	Bench press	5 (61)	0.42	0.05, 0.78	0.03	0	0.40
	Elbow flexors	6 (104)	0.70	−0.34, 1.42	0.23	88	<0.0001
	Grip strength	1 (58)	1.17	0.78, 1.57	<0.0001	N	N
	MVC	5 (133)	0.41	−0.09, −0.92	0.10	70	0.009
	PV	7 (82)	0.90	0.18, 1.62	0.01	77	0.0002
	P (W)	4 (44)	1.87	0.22, 3.53	0.03	89	<0.0001
Exercise intensity	≤30%1RM	14 (292)	0.65	0.27, 1.03	0.0007	77	<0.0001
	40%–70% 1RM	9 (112)	1.31	0.61, 2.01	0.0002	81	<0.0001
	Maximal effort	6 (78)	0.21	−053, 0.95	0.57	78	0.0003
Compressive strength	≤40% AOP	3 (64)	0.52	−0.36, 1.40	0.25	82	0.003
	40%–60% AOP	4 (107)	0.44	−0.15, 1.03	0.14	71	0.02
	≥60% AOP	21 (311)	0.83	0.43, 1.23	<0.0001	80	<0.0001

Regarding exercise intensity subgroup analysis, the heterogeneity of the three groups was 77%, 81%, and 78%, respectively. Slight increase in heterogeneity was observed within the 40%–70% 1RM group (*I* ^2^ = 81%) compared to the overall combined effect (*I* ^*2*^ = 80%). The 40%–70% 1RM group showed the highest effect size and statistical significance (*SMD* = 1.31, *p* = 0.0002), suggesting that upper limb BFR exercise at this intensity significantly induced PAP.

Analysis of pressure intensity subgroups showed heterogeneity of 82%, 71%, and 80% for the three groups, respectively. An increase in heterogeneity was noted within the 40% AOP and below group (*I* ^*2*^ = 82%) compared to the overall combined effect (*I* ^*2*^ = 80%). Among them, the ≥60% AOP group exhibited the highest effect size and statistical significance (*SMD* = 0.83, *p* < 0.01), indicating that BFR training at pressure intensities of ≥60% AOP significantly induced PAP.

### 3.6 Sensitivity analysis

Sensitivity analysis was conducted by excluding individual studies from each group to assess the heterogeneity of the included literature.

Table 4 illustrates that the combined effect size of BFR on upper limb muscle activation was [*SMD* = 0.89, 95% *CI* (0.21, 1.58), *p* = 0.01]. After removing individual studies, the range of combined effect *SMD* was between 0.64 and 1.06, with heterogeneity *I* ^*2*^ ranging from 90% to 92%, and all *p*-values were less than 0.05. No single study threatened the results of the meta-analysis, indicating good stability.

**TABLE 4:** Combined effects of upper limb muscle activation after excluding individual studies.

Study	*SMD*	95%*CI*	P (Merge effect)	I2 (%)
Ahmadi, 2021	1.00	0.30, 1.71	0.005	91
Bradley, 2024	0.64	0.00, 1.29	0.005	90
Buckner, 2018	0.97	0.24, 1.70	0.009	92
Che, 2022	0.94	0.21, 1.67	0.01	92
Dankel, 2017	0.92	0.18, 1.66	0.01	92
Dankel, 2017	0.96	0.24, 1.68	0.009	92
Lei, 2023	0.92	0.19, 1.65	0.01	92
Lin, 2017	0.95	0.22, 1.67	0.01	92
Jessee, 2017	0.85	0.11, 1.58	0.02	92
Sun, 2020	1.02	0.32, 1.72	0.004	91
Roehl, 2023	0.66	0.01, 1.31	0.03	91
Vivian, 2019	0.84	0.10, 1.58	0.03	91
Wang, 2023	0.92	0.19, 1.65	0.01	92
Yasuda, 2015	1.06	0.38, 1.73	0.002	91
Zhang, 2021	0.94	0.20, 1.69	0.01	92
Zhao, 2023	0.74	0.06, 1.42	0.03	91
Overall	0.89	0.21, 1.58	0.01	92

Table 5 shows that after excluding individual studies on the impact of single BFR training on PAP, the range of *SMD* was between 0.65 and 0.78, with heterogeneity *I* ^*2*^ ranging from 77% to 80%, and *p* < 0.0001. Compared to the overall combined effect size [*SMD* = 0.57, 95% *CI* (0.33, 0.82), and *p* < 0.00001], the study results remained unchanged, indicating good stability.

**TABLE 5:** PAP merger effect after excluding individual studies.

Study	*SMD*	95%*CI*	P (Merge effect)	I2 (%)
Ahmadi, 2021	0.76	0.44, 1.08	<0.0001	80
Bradley, 2024	0.70	0.38, 1.02	<0.0001	80
Buckner, 2018	0.77	0.45, 1.09	<0.0001	79
Henrique, 2019	0.73	0.40, 1.05	<0.0001	80
Serrano, 2023	0.73	0.41, 1.06	<0.0001	80
Serrano, 2023	0.71	0.39, 1.04	<0.0001	80
Li, 2022	0.74	0.41, 1.06	<0.0001	80
Linero, 2021	0.66	0.36, 0.97	<0.0001	77
Jessee, 2017	0.71	0.39, 1.04	<0.0001	80
Mendonca, 2018	0.76	0.43, 1.09	<0.0001	78
Wilk, 2020	0.73	0.41, 1.05	<0.0001	80
Wilk, 2020	0.68	0.36, 0.99	<0.0001	79
Wilk, 2022	0.69	0.38, 1.01	<0.0001	80
Wilk, 2022	0.72	0.39, 1.04	<0.0001	80
Wilk, 2020	0.75	0.43, 1.07	<0.0001	80
Wilk, 2020	0.75	0.43, 1.08	<0.0001	80
Wilk, 2021	0.65	0.35, 0.95	<0.0001	78
Wilk, 2021	0.65	0.35, 0.94	<0.0001	77
Rodrigues, 2023	0.75	0.43, 1.07	<0.0001	80
Salagas, 2022	0.73	0.40, 1.05	<0.0001	80
Sun, 2020	0.78	0.47, 1.09	<0.0001	79
Thiebaud, 2014	0.75	0.42, 1.07	<0.0001	80
Vivian, 2019	0.71	0.38, 1.04	<0.0001	79
Zhang, 2023	0.75	0.43, 1.07	<0.0001	80
Zhang, 2023	0.76	0.44, 1.08	<0.0001	80
Zhang, 2023	0.76	0.44, 1.08	<0.0001	80
Zhang, 2021	0.75	0.42, 1.08	<0.0001	80
Zhao, 2023	0.72	0.40, 1.05	<0.0001	80
Overall	0.73	0.41, 1.04	<0.0001	80

The same literature name refers to different research results included in the same literature.

### 3.7 Publication bias

**FIGURE 5:**
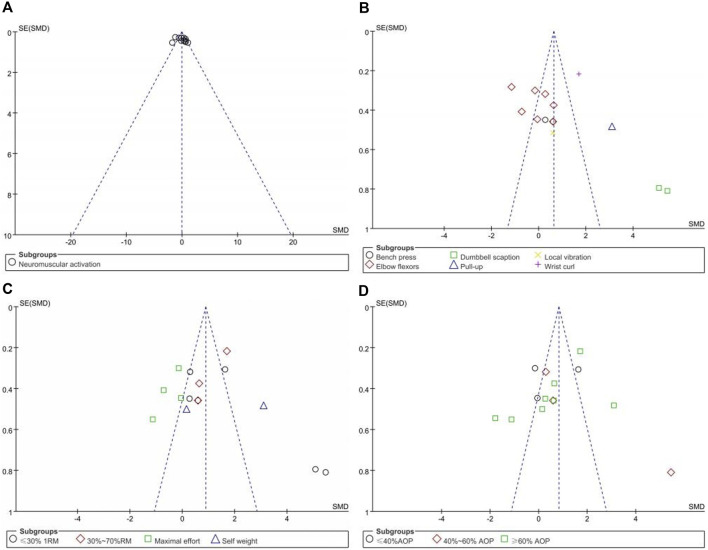
Funnel plots of Neuromuscular Activation:**(A)** Combine funnel chart; **(B)** Exercise mode; **(C)** Exercise intensity; **(D)** Compressive strength.

**FIGURE 6:**
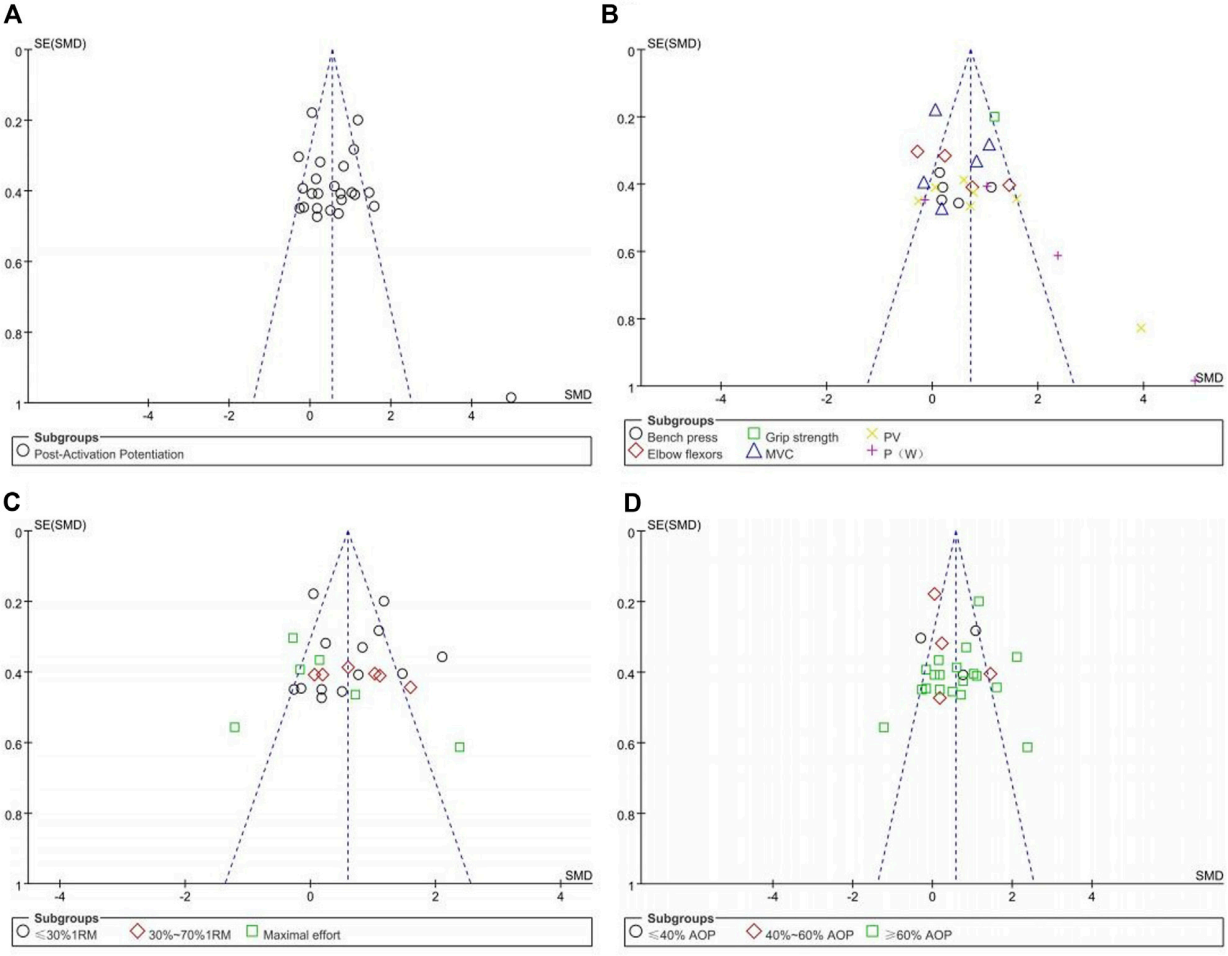
Funnel plots of Post-Activation potentiation:**(A)**; **(B)** Testing methods; **(C)** Exercise intensity; **(D)** Compressive strength.

The asymmetry of the funnel plots examining the publication bias for the subgroup analysis of the effects of upper limb BFR training on muscle activation and PAP is depicted in Figures 5, 6. Symmetrical funnel plots indicate the absence of publication bias. Furthermore, Egger’s test conducted on the funnel plots yielded *p*-values all greater than 0.05, suggesting no publication bias in the literature included in this study.

## 4 Discussion

### 4.1 BFR training-induced muscle activation in the upper limbs

Previous studies have indicated significant effects of BFR training on enhancing upper limb muscle circumference, strength, and endurance (Amani et al., 2019). However, the optimal training protocol for upper limb application of BFRT requires further investigation. This study conducted heterogeneous grouping of exercise, intensity, and BFR prescription based on collected data to evaluate the impact of BFRT on upper limb muscle activation.

During BFR, restriction of blood flow proximally in the limb leads to congestion of distal muscles, resulting in localized limb hypoxia and accumulation of lactate, thereby recruiting additional fast-twitch muscle fibers for movement (Yasuda et al., 2006). Meta-analysis results demonstrate a positive overall effect size across 21 studies (*p* < 0.05), indicating that BFR training significantly increases muscle activation in the upper limbs. Sensitivity analysis revealed no significant change in heterogeneity or combined effect size after excluding any individual study, suggesting consistent results across the included studies.

Previous research has found a correlation between muscle activation induced by BFRT and recruitment of more type II (fast-twitch) fibers (Yasuda et al., 2015b). The control groups included in the literature reviewed in this study all involved resistance exercises without pressure. Therefore, compared to non BFRT exercise, it is speculated that BFRT may promote the recruitment of more type II muscle fibers. However, due to the high heterogeneity observed in the study results, subgroup analysis was performed based on different study characteristics.

#### 4.1.1 Exercise mode

The within-group heterogeneity under different exercise modes was significantly reduced compared to the overall combined heterogeneity, indicating the need for future studies to classify exercise modes. The results showed that: 1) The dumbbell scaption exercise mode is more effective in eliciting upper limb muscle activation. 2) No significant effect was observed in the elbow flexor movement pattern, which may be attributed to higher heterogeneity in the literature or varying levels of fatigue generated by different planning schemes. (*I* ^2^ = 76, *p* < 0.01).

For Elbow flexors, 5 out of 9 studies showed negative effect sizes. The author noted that these 5 studies had exercise intensities of exhaustive training compared to other studies. Therefore, a second subgroup analysis was conducted based on different exercise intensities for this study.

#### 4.1.2 Exercise intensity

The study found that BFRT at different exercise intensities yields different results in upper limb muscle activation. Specifically, BFRT at ≤30% 1RM significantly enhances upper limb muscle activation, while maximal effort BFRT negatively affects upper limb muscle activation. When designing a training program for upper limb muscle activation, trainers might avoid using exhaustive BFRT with loads above 40% 1RM. Instead, they could focus on lighter loads and potentially alternative methods to achieve the desired muscle activation.

#### 4.1.3 Compressive strength

After conducting subgroup analysis based on different compressive strengths for upper limb muscle activation, we found that within-group heterogeneity was high, and the differences within groups were not significant. This result may reflect that within the range of compressive strengths selected in this study, there was no significant impact on upper limb activation. This could be due to several factors.

Firstly, compressive strength may not be the sole factor influencing upper limb muscle activation. Compared to lower limb muscles, upper limb muscles typically have fewer muscle fibers and lower blood supply, which may limit the impact of blood flow restriction training on upper limb activation (Thomas et al., 2020). Therefore, the response of upper limb muscles to different compressive strengths may be weaker, which could be one reason why significant effects were not observed in this study.

Secondly, individual differences may also influence the results. Physiological characteristics, exercise experience, and muscle tissue properties of different individuals may lead to varied responses to compressive strength. Additionally, other individual factors such as pain perception and psychological state may also affect muscle activation. Finally, relevant studies have found that numerous cuff features may impact BFR exercise (Rolnick et al., 2023).

### 4.2 Possible mechanism of inducing PAP by BFR training

As a physiological phenomenon characterized by a sudden increase in explosive strength, PAP is typically believed to be associated with H-reflex potentiation, changes in muscle fiber pennation angle, muscle acidification, and increased excitability of nerves under exercise stimulation, leading to recruitment of a greater number of motor units (Hamada et al., 2000). This phenomenon aligns with the results of the aforementioned studies on the effects of BFRT on upper limb muscle activation. Cleary (Cleary Christopher and Cook Summer 2020) also found that after BFR training, muscle fibers not only significantly increased in strength but also reached their highest levels of electromyographic amplitude. Our study demonstrated that upper limb BFRT had a positive impact on PAP, which is consistent with some earlier studies, further confirming the effectiveness of BFRT in improving muscle function.

Despite the positive results of our study, the heterogeneity was still significant (*I* ^*2*^ = 80%), indicating the need for further research to delve into the mechanisms of action of upper limb blood flow restriction training and its applicability in different populations and sports. Through subgroup analysis of PAP, we can better understand and harness the potential of upper limb BFRT, providing more precise and effective guidance for athlete training and performance.

#### 4.2.1 Testing methods

Different testing methods showed variations in the impact of BFRT on PAP. Firstly, the bench press group exhibited the highest homogeneity (*I* ^2^ = 0%), indicating more consistent results in bench press testing. In contrast, the Elbow flexors and maximum output power groups had higher levels of within-group heterogeneity (*I* ^2^ = 88% and *I* ^2^ = 89% respectively), suggesting greater variability in the observed effect sizes with these testing methods. Specifically, in our study, the bench press, P (W), and P (V) groups all showed statistically significant effects (*p* < 0.05) following upper limb blood flow restriction training. P (W) and P (V) represent the maximum power and velocity of the bench press respectively, indicating a significant enhancement in subjects’ explosiveness during the bench press motion after upper limb BFRT. These findings are consistent with previous research (Wilk et al., 2021), further validating the positive impact of upper limb blood flow restriction training on PAP.

However, it's important to note that differences exist between different testing methods, which could be attributed to the characteristics of the testing methods themselves and individual variations among the trainees. The bench press, as a common upper body strength training exercise, has a more stable and consistent movement pattern, thus exhibiting higher homogeneity in studies. On the other hand, maximum velocity and maximum power may be influenced by a greater number of factors, leading to higher heterogeneity in results. In summary, the differences in the impact of upper limb blood flow restriction training on PAP across different testing methods may reflect the inherent characteristics of the testing methods.

#### 4.2.2 Exercise intensity

Subgroup analysis based on different exercise intensities revealed that upper limb blood flow restriction training (BFR) significantly influenced the Potentiation after Activation (PAP) within the intensity range of 40%–70% of 1RM. Specifically, we observed the highest effect size in the training groups within this intensity range, which was statistically significant (*SMD* = 1.31, *p* = 0.0002), indicating that BFR exercises at this intensity level can significantly induce PAP.

For traditional resistance training, effective enhancement of muscle absolute strength typically requires intensities of ≥70% of 1RM (Kanehisa et al., 1989). Although recruiting more muscle fibers is believed to enhance muscle strength (Boullosa et al., 2013), studies by Serrano-Ramon et al. (2023) combining BFR training with exercises at 60% of 1RM for bench press found similar responses to heavy-load exercises. This finding is consistent with our results. Regarding exercise intensities of ≤30% of 1RM, although they also induce PAP, the effect size is lower (*SMD* = 0.65, *p* = 0.0007), which may be attributed to the excessively low intensity of the exercises. Low-intensity training may not sufficiently stimulate upper limb muscle fibers (Shono et al., 2002). Therefore, exercise intensity within the range of 40%–70% of 1RM has been supported as an effective method for inducing PAP, providing athletes and coaches with a simple and efficient training regimen.

#### 4.2.3 Compressive strength

Compressive strength plays a crucial role in inducing Potentiation after Activation (PAP). Specifically, our results indicate that higher intensities, especially those ≥60% of AOP, are more effective in inducing PAP compared to lower intensities. This underscores the need to carefully consider compressive strength when designing and implementing blood flow restriction protocols to optimize their effects on enhancing muscle performance.

Studies have shown that BFRT at lower compression intensities fails to elicit a stress response in the body (Michal et al., 2022). Additionally, the increased heterogeneity observed within the low-intensity groups suggests significant differences or variations in results within this range of compression intensities. Future research should aim to elucidate the mechanisms underlying the intensity-dependent effects of blood flow restriction training on PAP and explore potential moderating factors contributing to the observed heterogeneity. Overall, these findings provide valuable insights for practitioners and researchers in designing and interpreting blood flow restriction training protocols aimed at inducing PAP and enhancing muscle performance.

### 4.3 Study limitations

In the quality assessment of the studies, some literature had a higher risk of bias in blinding due to ethical requirements for human experiments. Additionally, deficiencies in study design were noted in some literature, as they did not clearly describe specific operational procedures and control variables during the experiments, potentially leading to significant heterogeneity in study results. Among the included literature, there may be limitations in sample characteristics, such as age, gender, and level of physical activity, which could influence the study outcomes. A significant limitation is the large differences in applied pressures, protocols and populations used in the studies. This makes drawing strong conclusions very challenging. Future research could consider addressing these sample differences to more comprehensively evaluate the effectiveness of upper limb blood flow restriction training.

## 5 Conclusion

Upper limb blood flow restriction training (BFRT) can induce muscle activation and post-activation potentiation (PAP), particularly when conducted at higher compressive strengths (≥60% AOP) and moderate exercise intensities (40%–70% 1RM). Furthermore, BFRT enhances explosive force indicators, especially in the bench press exercise.
